# Modified ELISA for Ultrasensitive Diagnosis

**DOI:** 10.3390/jcm10215197

**Published:** 2021-11-07

**Authors:** Naoko Tsurusawa, Jyunhao Chang, Mayuri Namba, Daiki Makioka, Sou Yamura, Kanako Iha, Yuta Kyosei, Satoshi Watabe, Teruki Yoshimura, Etsuro Ito

**Affiliations:** 1 Department of Biology, Waseda University, Tokyo 162-8480, Japan; kitastone7n@ruri.waseda.jp (N.T.); jyunhao@suou.waseda.jp (J.C.); myrnmb@asagi.waseda.jp (M.N.); mkokdik-3434@ruri.waseda.jp (D.M.); souyamura@toki.waseda.jp (S.Y.); kanako-anastasia@asagi.waseda.jp (K.I.); yuta.18-baseball@asagi.waseda.jp (Y.K.); 2 Waseda Research Institute for Science and Engineering, Waseda University, Tokyo 169-8555, Japan; s.watabe@kurenai.waseda.jp; 3 School of Pharmaceutical Sciences, Health Sciences University of Hokkaido, 1757 Kanazawa, Ishikari-Tobetsu 061-0293, Hokkaido, Japan; yosimura@hoku-iryo-u.ac.jp; 4 Graduate Institute of Medicine, School of Medicine, Kaohsiung Medical University, Kaohsiung 80756, Taiwan

**Keywords:** diagnosis, infectious disease, lifestyle-related disease, ultrasensitive ELISA, thio-NAD cycling

## Abstract

An enzyme-linked immunosorbent assay (ELISA) can be used for quantitative measurement of proteins, and improving the detection sensitivity to the ultrasensitive level would facilitate the diagnosis of various diseases. In the present review article, we first define the term ‘ultrasensitive’. We follow this with a survey and discussion of the current literature regarding modified ELISA methods with ultrasensitive detection and their application for diagnosis. Finally, we introduce our own newly devised system for ultrasensitive ELISA combined with thionicotinamide adenine dinucleotide cycling and its application for the diagnosis of infectious diseases and lifestyle-related diseases. The aim of the present article is to expand the application of ultrasensitive ELISAs in the medical and biological fields.

## 1. Introduction

The enzyme-linked immunosorbent assay (ELISA) is an easy-to-use method for detecting proteins (e.g., pathogenic proteins, antigens, and antibodies) on the basis of an enzyme reaction [1]. The potential applications of this method depend on the specificity and strength of the antigen–antibody reaction being evaluated. Due to the limitation of ELISAs requiring the use of a purified antigen and an extraordinarily functional antibody, some researchers strongly propose that other methods be used, such as liquid chromatography–mass spectrometry (LC-MS) [2,3,4,5,6,7,8,9,10,11] and various lab-on-a-chip methods [12,13,14,15,16,17,18,19,20,21,22,23]. A recent trend in LC-MS development for diagnosis is to focus on the detection of drugs and biomarkers, including peptides and steroids, owing to difficulties in preparing suitable antibodies against these small molecules, whereas lab-on-a-chip–based platforms are thought to offer easier sample preparation, chemical manipulation and reaction, and high throughput using various novel detection techniques with, sometimes, label-free detection.

Beyond this limitation, however, the ELISA method has many advantages, including simplicity, practicality (special and expensive apparatuses are not necessary if the ELISA is used as a colorimetric method), rapidness, and reasonable cost [24]. The remaining challenge for conventional ELISA is to increase the detection sensitivity. The sandwich-type ELISA, in which two antibodies recognizing two different epitopes of a target protein are used for immobilization on a solid surface and for specific capture, is suitable for quantitative detection. Many studies report high detection sensitivity using ELISA with some modifications, thus providing ultrasensitive ELISA methods [25].

In the present review article, we first define what we mean by ‘ultrasensitive’, which is often misinterpreted, leading to confusion. Next, we survey and discuss the current literature about ultrasensitive ELISA and its applicability for diagnosis. Lastly, we introduce our own newly devised system for an ultrasensitive ELISA coupled with thionicotinamide adenine dinucleotide (thio-NAD) cycling and its application to diagnosis for infectious diseases and lifestyle-related diseases.

## 2. What Does ‘Ultrasensitive’ Mean?

The strongest requirement for conventional ELISA is the ability for ultrasensitive detection [26,27,28]. The term ‘ultrasensitive’, however, is often misinterpreted. One reason for this is the comparison with the limit of detection (LOD) in polymerase chain reaction (PCR). PCR can amplify nucleic acids, resulting in the detection of a small number of DNA or RNA copies. For example, the LOD of real-time PCR is a few copies/assays for various nucleic acids [29]. However, the LOD cannot be compared between real-time PCR and protein measurement systems in which proteins are not amplified. Thus, it is inaccurate and misleading to state that the LOD of protein measurement systems is inferior to that of PCR and that the word ‘ultrasensitive’ should not be used for protein measurements.

A second reason for the misinterpretation of ‘ultrasensitive’ originates from comparison of the LOD among various other methods for protein measurements. As described above, many techniques can be used to quantify target proteins, including ELISA, LC-MS, and lab-on-a-chip methods [30]. Whereas the method with the lowest LOD might be considered an ‘ultrasensitive’ method and superior to others, it is not appropriate because it is useless to compare methods solely on the basis of detection limits without also considering the need for a special apparatus or a special room, as well as the cost. That is, the term ‘ultrasensitive’ does not imply a specific value. So, then, what is ‘ultrasensitive’?

J. Justin Gooding from the University of New South Wales, Australia, recently addressed this question in detail [31]. The term ‘ultrasensitive’ is used differently between chemists and biologists. To define the term in an interdisciplinary manner, he defined an ultrasensitive bioanalytical sensor as “a biosensor with sufficient sensitivity and low background to allow subpicomolar detection limits”. He also explained that “this is a compromise between the chemical and biological meaning of sensitivity that acknowledges the link between the chemical meaning of a detection limit, and sensitivity as it relates to the background signal”. An LOD of 10^−13^ moles/L or smaller can be referred to as ‘ultrasensitive’ detection. If we use a 96-well microplate for ELISA, one assay volume is generally equal to 100 μL. Thus, 10^−13^ moles/L is equivalent to 10^−17^ moles/assay, which might be challenging to achieve. Our own ELISA method, however, can detect proteins at zeptomolar detection limits, i.e., 10^−21^ moles/assay [32], and thus, we believe that our system can be classified as an ‘ultrasensitive’ ELISA.

## 3. Recent Advances in Modified ELISA Methods to Realize Ultrasensitive Diagnosis

To increase the detection sensitivity of ELISA, various strategies have been employed. In this section, we introduce the state-of-the-art technology for ELISA.

### 3.1. Digital ELISA

**Figure 1:**
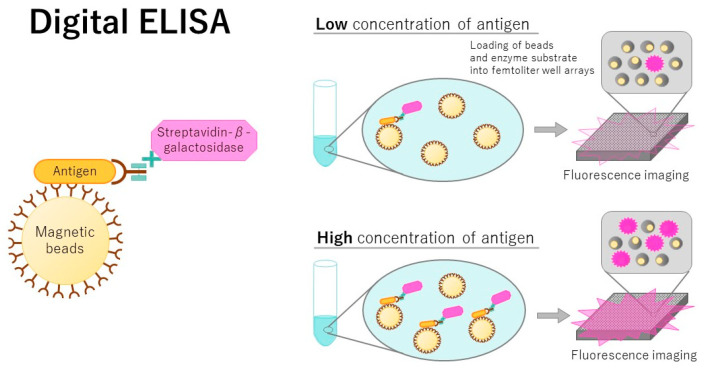
Scheme of a digital ELISA. The left panel shows that antibody-conjugated magnetic beads are first used to capture single molecules of the target protein (i.e., antigen), and the protein-bead complexes are labeled with a second antibody-conjugated enzyme. The right panel shows a case of low antigen concentration and a case of high antigen concentration. In the tube, first, antibody-conjugated magnetic beads, an antigen, and a second antibody-conjugated enzyme are sequentially mixed. Then, the bead complexes are assessed using a precision-fabricated, femtoliter-volume microwell array capable of capturing one bead per well. After adding the substrate as a fluorophore, fluorescence images of a small section of the well array are obtained. The concentration of protein in bulk solution is correlated with the percentage of beads that carry a protein molecule [33].

Digital ELISA was developed approximately 10 years ago as an ultrasensitive detection technique (Figure 1) [33]. When the enzymatic reaction is achieved in femtoliter-sized microwells, the accumulated product is detectable at very low concentrations of the target, thereby achieving single molecule detection. Using a digital ELISA, a femtomolar concentration of recombinant nucleoprotein for influenza A virus was detected [34]. A digital ELISA using bifunctional fluorescence magnetic nanospheres with gold nanoparticles (AuNPs) was applied to detect avian influenza virus [35]. The LOD was 7.8 fg/mL, which was promising for single biomolecular detection. Many improvements have been described for digital ELISA. Akama and colleagues developed a highly sensitive detection method modified from a digital ELISA without washing and signal amplification steps, and they called it a digital homogeneous non-enzymatic immunosorbent assay [36]. This assay detected 0.093 pg/mL of a prostate-specific antigen, whereas a commercialized highly sensitive ELISA detected 0.84 pg/mL and digital ELISA detected 0.055 pg/mL. The lack of washing and amplifications steps in the detection method developed by Akama and colleagues is a remarkable feature.

The digital ELISA was modified to count single molecules. This technique is the dropcast single molecule assay [37], in which beads are dropcast onto a microscope slide and dried in a monolayer film for digital signal readout. This assay detects attomolar levels of proteins. Digital ELISA was combined with a quantitative viral outgrowth assay for the ultrasensitive detection of human immunodeficiency virus (HIV)-1 p24 proteins [38] and could achieve femtogram detection of p24 in contrast to the picogram limitations of a traditional ELISA. A digital ELISA was also used for the simultaneous detection of spike and nucleocapsid proteins of the severe acute respiratory syndrome coronavirus 2 (SARS-CoV-2) [39]. SARS-CoV-2 is a virus causing the coronavirus disease 2019 (COVID-19) infection. This assay combined the magnetic-bead-encoding technology and the ultrasensitive detection capability of a single molecule assay. The LOD of spike and nucleocapsid proteins was 20.6 and 69.8 fg/mL, respectively.

### 3.2. Application of AuNPs to ELISA

**Figure 2:**
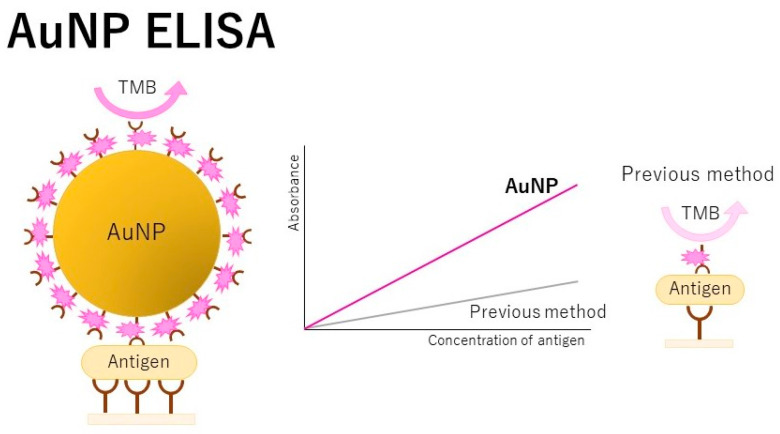
Scheme of ELISA using gold nanoparticles (AuNPs). This ELISA has unique structural, electronic, magnetic, optical, and catalytic properties. The use of AuNPs allows the attachment of a multiple enzyme system that can generate an amplified optical signal while keeping low background signals [40]. TMB is tetramethylbenzidine.

AuNPs are also used for immunosensing proteins in sandwich-type assays (Figure 2) [40]. The use of AuNP labels increases the surface stress on the immunocomplex mass, allowing for an important increase in the sensitivity of electromechanical assays. For example, a detection method for the hepatitis B virus was developed using an invader-assisted ELISA assay [41]. The invader reactions amplified the biotinylated oligonucleotide specific to the antigen, resulting in detection of the antigen. Oligonucleotide probes and modified AuNPs were coupled with the invader assay. The detection sensitivity was 10^−11^ g/mL, which could even be observed with the naked eye. Avian influenza virus A was ultrasensitively detected with an ELISA using AuNPs and magnetic beads [42]. Viruses present at 25 pg/mL were detected by the naked eye. Moreover, an enhanced fluorescence ELISA was developed based on human alpha-thrombin triggering fluorescence turn-on signals [43]. In this system, the detection antibodies and human alpha-thrombin were labeled with AuNPs and a bisamide derivative of Rhodamine_110_ with fluorescence quenched serving as the substrate of human alpha-thrombin. α-Fetoprotein and hepatitis B virus surface antigens were detected at levels of 10^−8^ ng/mL and 10^−4^ IU/mL, respectively; these levels were 10^4^ times lower than those of the conventional fluorescence assays and 10^6^ times lower than those of the conventional ELISAs.

Techniques using AuNPs were also applied to detect many biomarkers. A plasmonic ELISA with AuNPs was used to detect *Mycobacterium tuberculosis* ESAT-6-like protein esxB, showing that the LOD of 0.01 μg/mL was detectable by the naked eye [44]. This technique may be important for the early diagnosis of tuberculosis. AuNPs are well assembled by polyamidoamine dendrimers, which are highly branched three-dimensional macromolecules with hydrophilic terminal groups (-NH_2_). The structural properties of polyamidoamine dendrimers contribute to the amplification of the signal in the immunoassay. Using this assay, human chorionic gonadotropin was detected at 0.03 IU/L [45]. Measuring human chorionic gonadotropin levels can be helpful for discriminating a normal vs. a pathologic pregnancy. A AuNP-based ELISA with an LOD of 0.9 pg/mL was applied to the nucleocapsid proteins in bunyavirus for severe fever with thrombocytopenia syndrome [46]. The combination of AuNPs and bio-conjugated magnetic nanochains enabled the development of a dynamic and pseudo-homogeneous ELISA [47]. Accurate and ultrasensitive detection of acute myocardial infraction biomarkers was also achieved using this assay.

### 3.3. Application of AgNPs to ELISA

The use of silver nanoparticles (AgNPs) also increases the detection sensitivity of ELISAs. For example, AgNPs are thought to be an ideal colorimetric indicator because of the high extinction coefficient and strong plasmon resonance absorption property, e.g., for achieving excellent detection performance in surface-enhanced Raman scattering detection. Using an AgNP technique, an LOD for alkaline phosphatase (ALP) was 0.003 U/L, and that of carbohydrate antigen 125, which is found on ovarian cancer cells, was 1.75 U/mL [48]. A combination of Au- and Ag-NPs was used to detect norovirus [49] with an LOD of 10.8 pg/mL, which is a 1000- and 100-fold higher sensitivity compared with the gold-immunoassays and the horseradish-peroxidase-based conventional ELISA, respectively.

### 3.4. Chemiluminescence ELISA

Chemiluminescence and electrochemiluminescence are also used for ELISA. A sandwich ELISA using a chemiluminescence substrate was applied to measure amyloid-β_42_ (Aβ_42_) in the plasma of patients with Alzheimer disease [50]. The LOD was 1 pg/mL, and this assay could measure Aβ_42_ levels in both patients with Alzheimer disease and normal subjects. The sensitivity of the chemifluorescence ELISA was enhanced using photooxidation-induced fluorescence amplification [51]. When this assay was applied to a commercial kit to detect Aβ_42_, it enhanced the detection sensitivity by a factor of more than 10. Electrochemiluminescence ELISA combined with an Au nanocluster technology was developed, and the LOD for tumor necrosis factor-α (TNF-α) was two orders lower than that using a conventional ELISA [52].

### 3.5. Application of Europium to Fluoroimmunoassay

The trivalent europium ion (Eu^3+^) is an attractive rare-earth metal due to its bright fluorescence when chelated by organic ligands. A fluoroimmunoassay using Eu^3+^ was developed for the quantitative analysis of saikosaporin a, which is a traditional Chinese medicine, and had an LOD of 0.006 μg/mL [53]. A time-resolved fluoroimmunoassay was established for the ultrasensitive detection of aflatoxin B_1_, which is a key factor causing liver cancer, using Eu^3+^-labeled IgG as a tracer [54]. The IC_50_ was 94.73 pg/mL and the LOD was 3.55 pg/mL.

### 3.6. Other Techniques to Realize Ultrasensitive ELISA

A unique approach was used by Hashida’s group [55]. This ultrasensitive assay is referred to as an immune complex transfer enzyme immunoassay, which is 100-fold more sensitive than conventional ELISAs. For example, it could detect glutamic acid decarboxylase autoantibodies in serum collected from patients with type 2 diabetes and is useful for predicting diabetic onset and early diagnosis. Another unique method is an ultrasensitive detection technique without an enzyme reaction. This detection technique uses an enzyme-free signal amplification based on gold vesicles encapsulated with Pd-Ir nanoparticles [56]. The LOD was at the femtogram/mL level, which was 1000-fold lower than that of conventional horseradish-peroxidase-based assays. Ultrasensitive detection was reported using nanolabeled high-index {*hk*0}-faceted platinum concave nanocubes [57]. Using this method, human-prostate-specific antigen, which is a prostate cancer biomarker, was detected at 0.8 pg/mL, a level lower than that detected by conventional ELISA. Furthermore, a temperature-responsive liposome-linked immunosorbent assay was reported [58]. This technique uses temperature-responsive liposomes containing a squaraine dye that exhibits fluorescence at the phase transition temperature of the liposomes. The LOD of prostate-specific antigen was 0.97 aM.

Two nanobodies targeting two distinct epitopes of the protein, one of which is used for capture and the other of which is fused with the firefly luciferase serving as a reporter, enabled researchers to develop a dual-epitope protein identification assay with a sensitivity of 10 pg/mL for soluble CD38 [59]. The soluble CD38 levels in plasma correlate with the proliferation of multiple myeloma cells, and this ultrasensitive assay is thought to be useful for diagnosing multiple myeloma and CD38-related diseases. For another type of ultrasensitive detection, a colorimetric readout was replaced with an electro-readout, which was based on label-triggered degradation of methylene blue [27]. The LOD of cancer antigen 125 was 0.048 mU/mL, which was six-fold more sensitive than that using conventional ELISA. Human-serum-albumin-based nanoparticles, which are capable of encapsulating horseradish peroxidase, were applied to the development of another ultrasensitive ELISA [26]. Using this ELISA, thioredoxin-1, which is a breast cancer biomarker, was measured in the range of 10 fM to 100 pM—10^5^ times more sensitive than conventional methods.

An on-chip optofluidic ELISA was developed and applied to detect a biomarker, vascular endothelial growth factor, with an LOD of 17.8 fg/mL [60]. This assay used an optofluidic chip technique integrated with directly printed, high-quality-factor polymer microlaser sensors and optical whispering-gallery-mode microcavities for an ultrasensitive ELISA. Another ultrasensitive ELISA was developed using horseradish peroxidase-loaded dendritic mesoporous silica nanoparticles modified with poly(amino acid) multilayers [28]. The LOD of this assay was 10^4^ times lower than that of a conventional ELISA. Progesterone, as a stress-affecting factor and a carcinogen, was measured in saliva using a new type of nanoflower [61]. This assay was developed as a multiple-catalysis ELISA using copper phosphate, Pt/IrO_2_ nanocomposites, streptavidin, and horseradish peroxidase, resulting in an LOD of 0.076 ng/mL. For faster and more ultrasensitive reactions, rolling circle amplification and enzymatic signal amplification were applied to an ELISA [62]. This assay was accomplished within 10 min, and the LOD for CEACAM-7 was 2.82 pg/mL, which was about 50 times lower than that of a traditional ELISA. Carcinoembryonic antigen-related cell adhesion molecule 7 (CEACAM-7) is a glycosylphosphatidylinositol-anchored glycoprotein that is preferentially expressed on the luminal surface of epithelial cells near the mouth of colonic crypts and on pancreatic ductal epithelial cells. The sensitivity for carcinoembryonic antigen as a cancer marker was improved six-fold in comparison with a conventional ELISA.

Another ultrasensitive assay is called a ‘cell ELISA’. A modified cell-based ELISA detected cancer cells using a colorimetric method [63]. In this assay, a folic-acid-functionalized in situ-grown MnO_2_ nanosheet/graphene oxide hybrid with oxidase-like activity was used instead of the anti-folate receptor antibody in a traditional cell-based ELISA. Twenty cancer cells were detected using a microplate reader, and 75 cells were identified by the naked eye.

We know a very famous method, which is called immuno-PCR, of increasing the detection limit of a conventional ELISA about 1000- to 10,000-fold [64]. In immuno-PCR, the detection enzyme in ELISA was replaced with a biotinylated reporter DNA bound to the antigen–antibody complex through a streptavidin-protein A fusion protein. Immuno-PCR combined the exponential signal amplification power of PCR with the versatility and flexibility of ELISA. The LOD of real-time immuno-PCR was 40 pg/mL of the anti-cancer drug rViscumin in human plasma [65].

In summary, all of these techniques achieved ultrasensitive detection of proteins (see Table S1). The concern regarding these methods, however, is the need for a special apparatus or complicated training for experimenters. A simpler method for medical practice, e.g., even under conditions lacking air conditioning or electricity, is required.

## 4. An Ultrasensitive ELISA Combined with thio-NAD Cycling

**Figure 3:**
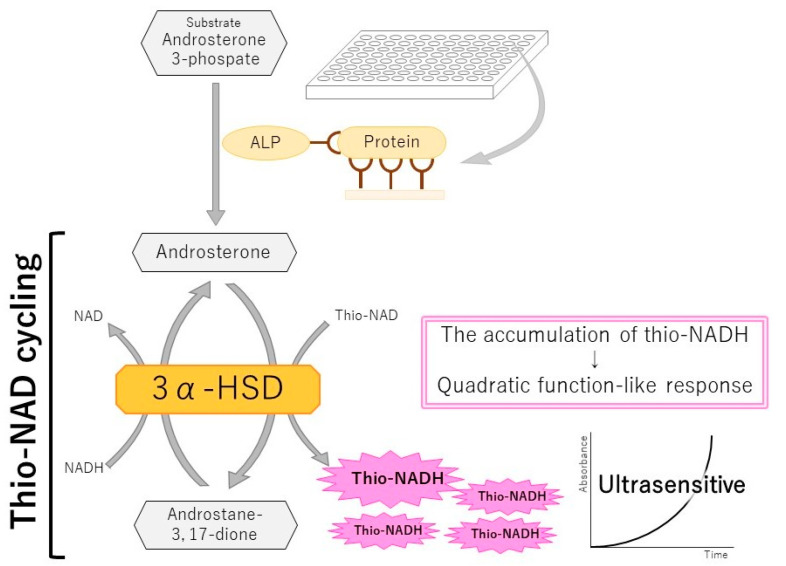
Scheme of an ultrasensitive ELISA with thio-NAD cycling. Both a conventional sandwich ELISA and a thio-NAD cycling assay produce signals as a linear function against time. When both assays are combined, the signals can be amplified in a quadratic-function-like response (e.g., triangle number) over time. This system comprises ALP linked with a secondary antibody against the target protein, an androsterone derivative as the first substrate, 3α-HSD as the enzyme for a thio-NAD cycling, and their coenzymes, such as NADH and thio-NAD. Thio-NADH accumulation is measured as absorbance at 405 nm [66].

In 2014, Watabe and Ito attempted to realize an ultrasensitive ELISA by combining a sandwich ELISA and thio-NAD cycling to achieve ultrasensitive, quantitative detection of proteins [66]. According to the interpretation of the term ‘ultrasensitive’ in Section 2, we called this system an ultrasensitive ELISA because ALP bound to the secondary antibody in a sandwich ELISA can be detected at the zeptomole (i.e., 10^−21^ moles) level [32]. A sandwich ELISA is used for the quantitative detection of a trace amount of proteins using two different antibodies specific to the target protein. Because proteins cannot be amplified, Watabe and Ito amplified the detectable signal for a trace amount of protein (Figure 3). In the next two sub-sections, we outline this ultrasensitive ELISA and describe its application for the diagnosis of various diseases.

### 4.1. Outline of an Ultrasensitive ELISA with thio-NAD Cycling

The major challenge for protein quantification is that proteins cannot be amplified like nucleic acids using a method such as PCR [29]. We hypothesized that we could, however, amplify the detection signal of specific proteins. For the production of a user-friendly diagnosis system, we designed a method for amplifying the signals produced by the antigen–antibody reaction in an ELISA [66]. How can ELISA signals be amplified?

The most suitable quantification technique for a trace amount of protein is a sandwich ELISA [67]. In the sandwich ELISA, the primary antibody is used to immobilize the target protein to a microplate, and the secondary antibody is conjugated to an enzyme (e.g., ALP) that converts the substrate to another form (Figure 3). A standard sandwich ELISA produces a color change in the substrate, resulting in a detectable signal. This signal increases in a linear fashion over time, and thus, the sensitivity is limited. This is a weak point of conventional ELISAs, and thus, there are many complaints about the low detection sensitivity. We considered that a substrate that is hydrolyzed by ALP could be amplified, which would provide the greatly desired signal amplification.

We identified another assay for detecting a trace amount of molecule that utilizes an enzyme cycling method to amplify the signal [68]. One of the co-authors of the Kato et al. paper [68], Lowry, wrote in 1980: “Enzymatic cycling provides a methodology for virtually unlimited amplification of analytical sensitivity. The most widely applicable cycling systems are those for NAD and NADP, since these can be used to increase the sensitivity of methods for a host of other substances” [69]. We thus considered that a combination of a sandwich ELISA and enzyme cycling could provide an ultrasensitive ELISA and be useful for early detection of disease-related biomarkers. Generally, in enzymatic cycling techniques, two enzyme reaction systems are used, in which each enzyme independently and cooperatively acts on the substrate–product pair in a different reaction direction. We pursued single-enzyme cycling using 3α-hydroxysteroid dehydrogenase (3α-HSD) [70,71,72,73] because the reaction system is simple and the detection system can be designed to be highly sensitive by developing steroidal substrates with high affinity for 3α-HSD and the labeled enzyme (e.g., ALP). The 3α-HSD catalyzes the substrate cycling between 3α-hydroxysteroid and its corresponding 3-ketosteroid in the presence of an excess amount of NADH and thio-NAD as the cofactors of 3α-HSD. When androsterone 3-phosphate is used as the first substrate (shown in Figure 3), androsterone corresponding to 3α-hydroxysteroid is produced by the hydrolysis of androsterone 3-phosphate via ALP, and androstane 3,17-dione corresponding to 3-ketosteroid is produced by catalyzation via 3α-HSD with NADH as a cofactor. Then, 3-ketosteroid (androstane 3,17-dione) returns to 3α-hydroxysteroid (androsterone) by catalyzation via 3α-HSD with thio-NAD as a cofactor (Figure 3). This series of reactions is called thio-NAD cycling [70,73]. In this reaction, thio-NAD is reduced to thio-NADH, and the accumulated thio-NADH is measured at an absorbance of 405 nm without any interference by the absorbance of NAD, NADH, and thio-NAD.

We combined these two assays, sandwich ELISA and thio-NAD cycling (Figure 3). This combination results in the phenomenon that the number of thio-NAD consumed in each of cycling steps increases linearly (i.e., one molecule, two molecules, three molecules, etc.) over time, whereas the thio-NADH accumulates in a quadratic-function-like fashion (i.e., triangle number) (Figure 3):

$$a\;\times \;b\;\times \;\sum_{k=1}^{n}k=a\;\times \;b\;\times \;\frac{n\left(n+1\right)}{2}$$

wherein *a* is the turnover ratio of ALP per minute, *b* is the cycling ratio of 3α-HSD per minute, and *n* is the measurement time in minutes.

### 4.2. Diagnosis of Infectious Diseases Using an Ultrasensitive ELISA with thio-NAD Cycling

The ultrasensitive ELISA with thio-NAD cycling has been used for the diagnosis of infectious diseases and lifestyle-related diseases. Among infectious diseases, we now face threats from COVID-19. To detect SARS-CoV-2, which causes COVID-19, its two major proteins were recently detected using the ultrasensitive ELISA with thio-NAD cycling [74]. One of the two proteins is a spike protein [75,76]. The LOD for the spike protein was 10^−18^ moles/assay. If the virus has approximately 25 spike proteins on its surface, this value corresponds to 10^−20^ moles of virus/assay. In other words, it could detect 10^4^ copies of the virus RNA/assay, which is almost the same as that of PCR assays because the average virus RNA load used for PCR assays is 10^5^ copies per oro- or naso-pharyngeal swab specimen [75]. To our knowledge, this is the first ultrasensitive antigen test for SARS-CoV-2 spike proteins. The other protein is a nucleocapsid protein [77]. The LOD for the nucleocapsid protein was 10^−17^ moles/assay. When UV-irradiated inactive SARS-CoV-2 was used, the minimum detectable amount of virus obtained was 10^4^ RNA copies/assay. The minimum detectable value was smaller than that of other antigen tests targeting SARS-CoV-2 nucleocapsid proteins, indicating the validity of this detection system for COVID-19 diagnosis. More recently, an ELISA with europium enhancement was proposed to detect the recombinant spike and nucleocapsid proteins from SARS-CoV-2 viruses [78]. Even though their method of determining the LOD was different from ours, our LOD is superior.

In addition to COVID-19, there are three other major infectious diseases of public health concern: tuberculosis, HIV/acquired immunodeficiency syndrome (AIDS), and malaria, which are serious threats to global health [79]. With regard to tuberculosis, live bacilli must be discriminated from dead bacilli for the assessment of therapeutic effects. PCR assays cannot make such a distinction. Live bacilli can be isolated using the gold-standard culture method, but this is a time-consuming technique. The ultrasensitive ELISA with thio-NAD cycling is useful for TB diagnosis because it can detect live bacilli with high sensitivity within hours [80]. When *Mycobacterium tuberculosis* is heated, only live bacilli secrete a specific protein, MPT64 [81,82]. The sputum obtained from tuberculosis patients was heated, and then MPT64 was detected. The LOD for MPT64 was 10^−19^ moles/assay, corresponding to 330 CFU/mL in the culture method, which was almost the same high detection sensitivity as the culture method. This ultrasensitive ELISA with thio-NAD cycling required only 5 h, resulting in a same-day diagnosis of tuberculosis and enabling drug efficacy monitoring.

For HIV detection, fourth-generation tests needed to detect both HIV-1/2 antibodies and the HIV-1 p24 antigen, which allowed the diagnosis to be made 1 to 3 weeks earlier than tests based on antibody alone. Furthermore, fifth-generation tests detected the same markers but could distinguish samples that were positive for HIV-1 p24 antigen and were specific for the HIV-1 and HIV-2 antibodies. Because of these technological advances, the window period for HIV detection has been reduced to approximately 18 days. To further decrease the window period, the ultrasensitive ELISA with thio-NAD cycling was applied for the detection of HIV-1 p24 [83]. The LOD for HIV-1 p24 was 10^−18^ moles/assay (i.e., ~0.15 IU/mL), which meets the qualification of 2 IU/mL established by the French Health Authority for a CE-marked HIV antigen/antibody assay in 2010. We are now devoting ourselves to the development of a specific detection system for malaria using the ultrasensitive ELISA with thio-NAD cycling.

### 4.3. Diagnosis of Lifestyle-Related Diseases Using an Ultrasensitive ELISA with thio-NAD Cycling

Insulin is a peptide hormone in the blood that serves as a signal of the fed state. Diabetes mellitus (DM) is a disease in which the control of insulin levels has failed. DM occurs in two forms—insulin-dependent, i.e., type 1 DM, and non-insulin dependent, i.e., type 2 DM. Patients with type 2 DM are often insulin-resistant and thus suffer from a relative insulin deficiency. In both cases, an ultrasensitive immunoassay approach for monitoring insulin is highly desirable and beneficial. The LOD of the ultrasensitive ELISA for a recombinant insulin was 10^−19^ moles/assay [66]. Another evaluation using the World Health Organization (WHO) international standard insulin reference (National Institute for Biological Standards and Control; Code 66/304) increased the LOD to 10^−16^ moles/assay [84]. The WHO insulin reference is purified from human pancreatic insulin, and it, or its equivalent, is widely used in commercial immunoassay kits. Thus, the assay sensitivity is highly dependent on the nature of the antigen and antibody used, making it difficult to compare performance characteristics obtained from different insulin immunoassays.

Adiponectin is a hormone secreted from adipocytes that demonstrates antidiabetic and anti-obesity activities. An increase in blood adiponectin levels is considered acceptable for good health; however, the normal urinary adiponectin levels were unknown because the urinary adiponectin levels in healthy subjects could not be determined using conventional ELISAs. The ultrasensitive ELISA with thio-NAD cycling was thus applied to detect a recombinant antigen of adiponectin and had an LOD of 10^−19^ moles/assay [80]. This adiponectin detection system was then applied to urine collected from healthy subjects and patients with DM [85,86]. The adiponectin level in the urine of healthy subjects was 3.06 ± 0.33 ng adiponectin/mg creatinine (mean ± SEM). On the other hand, the adiponectin level in the urine of DM patients was 14.88 ± 3.16 ng adiponectin/mg creatinine. The threshold between them could be set at 4.0 ng adiponectin/mg creatinine. Urinary adiponectin levels increase with the progression of chronic kidney disease [86].

## 5. Conclusions

Nucleic acids can be amplified with PCR, making it is easy to detect a small change in the amount of nucleic acids in cells and viruses. On the other hand, proteins, peptides, and many biomarkers cannot be similarly amplified. Therefore, detection techniques with high sensitivity are needed. While ELISA is a promising technique for this purpose, greater detection sensitivity is needed. Many variations of ELISA have been developed to achieve ultrasensitive detection, such as our ultrasensitive ELISA with thio-NAD cycling [87,88]. We hope that the present review article describing ultrasensitive ELISAs will be useful for not only protein researchers and engineers, but also for the development of diagnostic methods in the medical, biological, and industrial fields.

## Data Availability

Data are contained within the article.

## Supplementary Materials

The following are available online at https://www.mdpi.com/article/10.3390/jcm10215197/s1, Table S1: List of modified ELISAs. The ELISA principles, analytes, detection sensitivity, and reference numbers are shown.
