# Supplementary material for: Modified ELISA for Ultrasensitive Diagnosis
Source: J Clin Med. 2021 Nov 7;10(21):5197. doi: 10.3390/jcm10215197 (PMC8585087; doi:10.3390/jcm10215197)
Supplement: Supplementary file 1 [file jcm-10-05197-s001.zip › jcm-1440985-SI.pdf]

Table S1. List of modified ELISAs. The ELISA principles, analytes, detection sensitivity, and reference numbers are shown.

Category	Principle	Analyte	Detection sensitivity	Reference #
Digital ELISA	Fluorescence imaging for beads in femtoliter well arrays	PSA	14 fg/mL	33
Digital ELISA	Fluorescence imaging for beads in femtoliter well arrays	Recombinant nucleoprotein for influenza A virus	4 ± 1 fM (in buffer), 10 ± 2 fM (in 10-fold diluted nasopharyngeal swabs)	34
Digital ELISA	Bifunctional fluorescence magnetic nanospheres with AuNPs	H7N9 avian influenza virus	7.8 fg/mL	35
Digital HoNon-ELISA	Wash- and amplification-free digital immunoassay method	PSA	0.093 pg/mL	36
dSimoa	Dropcast single molecule assay	IL-10, IL-1β	19.2 aM (IL-10), 99.6 aM (IL-1β)	37
Digital ELISA viral outgrowth or DEVO assay	Digital ELISA combined with a quantitative viral outgrowth assay	HIV-1 p24	12 fg/mL	38
Digital ELISA	Magnetic bead-encoding technology	Spike and nucleocapsid proteins of SARS-CoV-2	20.6 fg/mL (spike protein), 69.8 fg/mL (nucleocapsid protein)	39
Invader assisted ELISA assay	Sandwich ELISA with DNA signal amplification	Recombinant HBV	2.4 × 10-11 g/mL (naked eye)	41
AuNP-based plasmonic sensors for ELISA	AuNP and plasmonic sensor	H7N9 avian influenza virus	25 pg/mL (naked eye)	42
Fluorescence enzyme-linked immuno-sorbent assay	Rhodamine, whose fluorescence was extinguished, as the substrate for HAT	α-Fetoprotein, hepatitis B virus surface antigen	10-8 ng/mL and 5 × 10-4 IU/mL	43
Plasmonic enzyme-linked immuno-sorbent assay	Plasmonic ELISA and biocatalytic cycle of intracellular enzymes to lead growth of AuNPs	Mycobacterium tuberculosis ESAT-6-like protein esxB (CFP-10)	0.01 μg/mL (naked eye)	44
ELISA with AuNP-PAMAM probe	Assemble of AuNPs induced by functional PAMAM	hCG	0.03 IU/L	45
AuNP-based ELISA	AuNP as a bridge between the detection antibody and HRP	Nucleocapsid protein of SFTSV	0.9 pg/mL	46
ELISA combining MNC and AuNP probe	MNCs facilitating liquid mixing and mass transfer; AuNPs co-immobilizing HRP and detection antibodies	Myoglobin (Mb), creatine kinase-MB (CK-MB), C-reactive protein (CRP)	147 pg/mL (Mb), 126 pg/mL (CK-MB), 53 pg/mL (CRP)	47
AgNPs with AA reducing Ag+ promoted by prereduction with $NaBH_4$	AA-mediated enhanced growth of AgNPs promoted by $NaBH_4$ as pre-reducing agent	ALP, carbohydrate antigen 125	0.003 U/L (ALP), 1.75 U/mL (carbohydrate antigen 125)	48
Enhanced colorimetric detection using in-situ growth of Ag shell on AuNPs	Enhanced colorimetric immunoassay for NoV detection for catalytic activity using in-situ growth of Au/Ag NPs	NoV-LP, NoV GII.4 and NoV GII.3 in fecal solution	10.8 pg/mL (NoV-LPs), 102 - 106 copies of viral RNA/mL (NoV GII.4 and NoV GII.3)	49
Chemi-luminescence ELISA	Chemiluminescence substrate	Aβ42	1 pg/mL	50
Sandwich ELISA with PIFA	Photooxidation-induced fluorescence amplification	Aβ42	enhanced the detection sensitivity by more than a factor of 10, few pg/mL	51
ECL-ELISA	High-performance ECL-ELISA with high-quantum-yield AuNC probe	TNF-α	36 fg/mL	52
Time-resolved fluoro-immuno-assay (TRFIA)	Fluoroimmunoassay using Eu3+	Saikosaponin a (SSa)	0.006 μg/mL	53
Time-resolved fluoro-immuno-assay (TRFIA)	Time-resolved fluoroimmunoassay using Eu3+-labelled IgG as a tracer	Aflatoxin B1 (AFB1)	3.55 pg/mL	54
ICT-EIA for GADA	Immune complex enzyme activity	Human glutamic acid decarboxylase 65 in serum	0.1 U/mL	55
Pd-Ir NPs@GVs based ELISA	Enzyme-free signal amplification technique based on gold	PSA	31 fg/mL	56

	vesicles encapsulated with Pd-Ir nanoparticles as peroxidase mimics			
HIF-PtCNC ELISA	HIF-Pt-CNC-labelled anti-PSA detection antibody as a signal probe	PSA	0.8 pg/mL	57
Temperature-responsive liposomes	Temperature-responsive liposomes containing SQR22 as a fluorescent detection probe	PSA	0.97 aM	58
DepID assay	Two nanobodies (Nbs) targeting two distinct epitopes of sCD38; one Nb acts as a capturer, and the other is fused with the firefly luciferase serving as a reporter to ensure sensitivity	Soluble CD38	10 pg/mL	59
Improved ELISA using electro-readout-mode based on label triggered degradation of methylene blue	Electro-readout mode using a system based on CS@hemin triggered degradation of MB	Cancer antigen 125 (CA125)	0.048 mU/mL	27
HEHPA based ELISA	Human serum albumin based nanoparticles capable of encapsulating excess HRP	Thioredoxin-1 (Trx1)	10 fM	26
On chip optofluidic ELISA	Polymer WGM microlaser-based optofluidic biochip for ELISA by printing high-quality polymer WGM microlaser sensors integrated into a microfluidic chip	VEGF	17.8 fg/mL	60
DSHP	Ultrasensitive ELISA system based on HRP-loaded dendritic mesoporous silica nanoparticles (DMSN) modified with poly(amino acid) multilayers	Mouse-IgG	0.667 fM	28
Heterologous multiple-catalysis ELISA	Powerful and sensitive multiple-catalysis ELISA due to the enhanced catalytic activity and stability of Pt/IrO2@SA@HRP nanoflowers	Progesterone (P4)	0.076 ng/mL	61
RELISA	RELISA integrating RCA reaction and improved colorimetric reaction induced by enriched enzyme	CEACAM-7 antigen	2.82 pg/mL	62
FA-MnO2/GO-based CELISA	Robust and sensitive sensing platform for the direct colorimetric detection of cancer cells based on a FA-MnO2/GO hybrid	Cancer cells	20 HeLa cells (plate reader), 75 cancer cells (naked eye)	63
Real-time immuno-PCR assay	Combination of highly sensitive immuno-PCR, employing standardized self-assembled DNA-protein conjugates as reagents, and the real-time PCR detection by means of the TaqMan principle	rViscumin, research antibody	40 pg/mL (rViscumin), 100 pg/mL (research antibody)	65
